# Long-term health-related quality of life improvements among patients treated with lurasidone: results from the open-label extension of a switch trial in schizophrenia

**DOI:** 10.1186/s12888-016-0879-5

**Published:** 2016-06-01

**Authors:** George Awad, Daisy Ng-Mak, Krithika Rajagopalan, Jay Hsu, Andrei Pikalov, Antony Loebel

**Affiliations:** Department of Psychiatry, University of Toronto, Toronto, ON Canada; Department of Psychiatry and Mental Health, Humber River Hospital, 1235 Wilson Avenue, 5th Floor, Toronto, M3M 0B2 ON Canada; Sunovion Pharmaceuticals Inc., Marlborough, MA USA; Sunovion Pharmaceuticals Inc., Fort Lee, NJ USA

**Keywords:** Health-related quality of life, Lurasidone, Long-term, Antipsychotic, PETiT, SF-12

## Abstract

**Background:**

Long-term improvement of health-related quality of life (HRQoL) in schizophrenia may improve adherence and reduce relapse and rehospitalization. This analysis examines long-term changes in HRQoL among patients with schizophrenia switched to lurasidone from other antipsychotics.

**Methods:**

Patients who completed an open-label 6-week switch study continued on lurasidone for an additional 24-weeks. HRQoL was measured using the self-reported Personal Evaluation of Transitions in Treatment (PETiT) scale and Short-Form 12 (SF-12) questionnaire. The PETiT assessed HRQoL via total and domain scores (adherence-related attitude and psychosocial functioning). The SF-12 assessed patients’ mental and physical component summary scores (MCS and PCS). Mean changes from the initial baseline were calculated at extension baseline and extension endpoint using analysis of covariance models. Analyses were further stratified by prior antipsychotic medication and responder status; responders were defined as having a ≥20 % improvement in Positive and Negative Syndrome Scale during the first 6-weeks of treatment.

**Results:**

The analysis included 144 patients with PETIT or SF-12 data who received ≥1 dose of lurasidone. Mean (standard deviation) PETiT total score improved significantly from 34.9 (9.3) at baseline to 39.5 (8.9) at extension baseline and 39.1 (9.0) at extension endpoint, representing improvements of 4.5 (7.9) and 5.1 (7.2) points, respectively (both *p* < 0.001). Significant improvements in adherence-related attitude and psychosocial functioning were observed at extension baseline and extension endpoint (all *p* < 0.001). Improvement in SF-12 MCS score was observed at extension baseline and endpoint, and PCS score at extension endpoint (all *p* < 0.01). Patients who switched from quetiapine and aripiprazole showed significant improvement of PETiT total score and adherence-related attitude at extension baseline and extension endpoint. In addition, patients who switched from quetiapine, risperidone, aripiprazole, or ziprasidone showed significant improvement in MCS scores from baseline to extension endpoint. Responders to lurasidone demonstrated greater improvement in PETiT total, psychosocial functioning, and MCS scores at extension baseline than nonresponders.

**Conclusions:**

After switching to lurasidone, patients with schizophrenia experienced HRQoL improvements that were sustained for an additional 24 weeks of treatment. Further study is warranted to understand the implications of these improvements in terms of employment, adherence, relapse, and rehospitalization.

**Trial registration:**

Clinical trials.gov identifier NCT01143090 (June 10th, 2010).

## Background

Schizophrenia is a severe, chronic, and debilitating disorder characterized by psychosis, behavioral dysfunction, and cognitive impairment. These manifestations not only impact the patient but also family, friends, and caregivers, as well as the healthcare system and society [1].

The prominent symptomatic manifestations of schizophrenia have been associated with considerable declines in health-related quality of life (HRQoL) [2–7]. HRQoL has been defined as the functional effect of a medical condition and its treatment on patient well-being [8–10]. This subjective and multidimensional outcome encompasses physical and occupational functioning, psychological state, social interactions, and somatic sensations [9]. HRQoL can be measured using a variety of general and disease-specific instruments that are typically patient-reported [11, 12]. For patients with schizophrenia, HRQoL and functioning are inversely associated with relapse and hospitalization rates, medication nonadherence, and treatment costs [13–18].

Treatment with atypical antipsychotics, the standard pharmacological treatment for schizophrenia, has led to improvements in HRQoL [19–22]. However, the impact of each drug in this class is variable, in part because of their unique clinical pharmacology and tolerability profiles [12, 23–25]. Identification of the optimal therapeutic regimen for each patient often requires switching between atypical antipsychotics to maximize relief from acute symptoms, improve long-term HRQoL and functioning, and minimize adverse effects [26, 27].

Given the importance of HRQoL in patients with schizophrenia, long-term studies can provide a broader picture of antipsychotic effectiveness in clinical practice. While results from clinical trials indicate that switching between antipsychotics can be performed in a relatively safe manner [20, 25–31], only a few studies have reported on the long-term (>6 months) effects of switching between treatments on HRQoL, patient attitude towards medication, or health status [25, 29, 32–36].

The efficacy, tolerability, and safety of lurasidone hydrochloride, a second-generation atypical antipsychotic approved for the treatment of adults with schizophrenia, have been demonstrated in published clinical trials [37–41] and summarized in review articles [42, 43]. A recent open-label clinical trial demonstrated that switching clinically stable yet symptomatic patients with schizophrenia or schizoaffective disorder to 6 weeks of treatment with lurasidone was well tolerated (i.e., low rates of patient discontinuation) and associated with improvements on the Positive and Negative Syndrome Scale [PANSS], the Clinical Global Impressions-Severity [CGI-S], and the Calgary Depression Scale for Schizophrenia [CDSS]) [44]. The 6-week trial also showed statistically significant improvements in overall HRQoL, adherence-related attitude, and psychosocial functioning and stabilization or improvement of health status using the self-reported Personal Evaluations of Transitions in Treatment (PETiT) scale and Short-form 12 (SF-12) instruments [45–47]. In a 24-week extension of the core 6-week switch trial, sustained efficacy was demonstrated on the PANSS, CGI-S, and CDSS, as was tolerability as measured by low rates [<10 %] of adverse events [AEs] and AE-related discontinuation and a lack of consistent, clinically relevant changes in metabolic outcomes [48]. The current follow-up analysis examines long-term HRQoL data for clinically stable yet symptomatic patients with schizophrenia switched to lurasidone in the 24-week extension period of the previously reported 6-week switch trial.

## Methods

### Study design

**Fig. 1:**
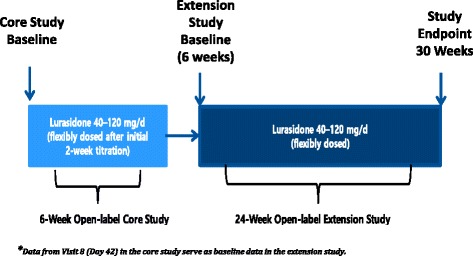
Design of the lurasidone open-label core and extension switch studies.* 144 of the 235 (61 %) patients included in the core 6-week study of HRQoL were included

The study design and primary outcomes of this clinical trial, which was conducted at 26 sites in the United States, have been previously published [44, 48]. In brief, the trial included patients who completed a 6-week core trial after being switched to lurasidone from their current antipsychotic therapy and continued on lurasidone in an open-label 24-week extension study (Fig. 1). Both the 6-week core and 24-week extension studies were approved by the Copernicus Group Independent Review Board and were conducted in accordance with Good Clinical Practices as required by the International Conference on Harmonization guidelines. All patients provided informed consent to participate in the study.

### Study patients

The trial included clinically stable but symptomatic adult outpatients aged ≥18 years who met the Diagnostic and Statistical Manual of Mental Disorders (DSM-IV-TR) criteria for a primary diagnosis of schizophrenia or schizoaffective disorder [44]. Patients were judged to be stable by the investigators based on the following criteria: a CGI-S score ≤4 at screening and baseline, a stable dose of pre-switch antipsychotic (s) for ≥28 days prior to screening, and no exacerbation of schizophrenia or schizoaffective disorder for ≥8 weeks prior to screening. Patients had at least a partial response to an antipsychotic and were stable at a dose consistent with product labeling. Unstable patients or those known to be treatment-resistant were excluded from the study. Patients were also required to have a clinically relevant reason for changing antipsychotics as determined by study investigators (i.e., insufficient efficacy and/or safety or tolerability concerns while on current medication despite attempts at optimization).

Patients who completed the 6-week core trial were continued on the same dose of lurasidone in the 24-week extension study (Fig. 1), providing a total of 30 weeks of data for analysis [48]. Lurasidone was flexibly dosed between 40 mg/day and 120 mg/day as clinically indicated, starting on Day 7, with dose adjustments occurring at weekly intervals as required. Additional details of the study design, patient inclusion criteria, and primary results of the 24-week extension study have been published by Citrome and colleagues [48].

HRQoL was evaluated in the current analysis using two subjective patient-reported outcome measures, the disease-specific PETiT scale [46] and the generic SF-12 Health Survey [47]. These measures were included as secondary endpoints in both the core and extension studies. Both of these measures have been used previously in the evaluation of HRQoL in patients with schizophrenia/schizoaffective disorder [20, 49–51].

### Outcome measures

#### PETiT scale

The PETiT scale is a validated, disease-specific, 30-item instrument designed to measure the impact of treatment on self-perceived subjective aspects of HRQoL in schizophrenia patients who are receiving antipsychotic drug therapy [46]. The scale assesses two highly relevant domains for schizophrenia: adherence-related attitude (includes six items reflecting adherence and feelings towards medication) and psychosocial functioning (24 items describing patient characteristics such as clarity, energy, concentration, functioning, sex drive, and memory). Psychosocial functioning can be assessed further within four subdomains: social functioning (four items on trust, confidence, and interactions), activity (seven items reflecting energy and ability to conduct daily tasks), cognitive (seven items on clarity, concentration, and communication), and dysphoria (six items on happiness, future, and self-esteem). Each item on the PETiT scale is assigned a rating of 0, 1, or 2, where 0 denotes worsened HRQoL and 2 denotes improved HRQoL. The PETiT total score ranges from 0 to 60, with higher scores denoting better patient HRQoL. The scale has high internal consistency (Cronbach’s alpha = 0.92) and high test-retest reliability (0.97; *P* < 0.001). Convergent validity has been demonstrated with the PANSS, the global assessment of functioning [GAF], and the quality of life scale [QLS] [46].

#### SF-12

The SF-12 survey is a generic 12-item instrument that is commonly used to measure the health status of patients with various diseases [47]. Scores on the SF-12 scale reflect changes in physical functioning, role limitations, health perceptions, bodily pain, vitality, social functioning, and mental health based on patient responses to 12 questions. The survey yields two summary measures of physical and mental health: the Physical Component Summary (PCS) and Mental Component Summary (MCS) scores. The SF-12 has been used in studies of patients with schizophrenia, where the MCS and PCS were found to be predictive of relapse and the MCS was predictive of remission [15, 52, 53].

### Analysis

The intent-to-treat (ITT) population in the 24-week extension study was used for the PETiT and SF-12 analyses. This population was defined as 1) patients who had received at least one dose of lurasidone, and 2) patients with non-missing values for PETiT or SF-12 scores at the core trial baseline and ≥1 post-baseline value. The HRQoL measures were collected at baseline in the core and extension studies and at Week 24 in the extension study. Patients who had only a core study baseline value but no extension study values (that is, all change-from-baseline values were missing), were excluded from the ITT analysis. Population sizes (N values) for PETiT and SF-12 scores varied across study time points because of variations in the availability of patient scores.

Similar to the analyses performed in the previous short-term evaluation of HRQoL [45], the current study examined mean changes in PETiT and SF-12 scores from study baseline to extension study baseline (6 weeks) and to extension study endpoint (end of the additional 24 weeks of treatment) using analysis of covariance (ANCOVA) models. Treatment and study center (pooled where necessary) were fixed factors and baseline value was a covariate. Changes were calculated for the PETiT scale total score, its domains and subdomains, and the SF-12 PCS and MCS scores for all patients switched to lurasidone. Changes in PETiT total and SF-12 scores were also analyzed by prior antipsychotic therapy for medications received by ≥9 % of subjects to ensure a minimal sample size for analysis.

The current study additionally evaluated PETiT (total and domains) and SF-12 scores by responder status. A “responder” was defined as a patient with a ≥20 % improvement on the PANSS total score during the initial 6-week study; a “nonresponder” was defined as a patient with a <20 % improvement on the PANSS during this timeframe. This response cutoff is commonly used in evaluations of patients with schizophrenia, with a ≥20 % early improvement being predictive of subsequent positive clinical outcomes [54–56].

## Results

### Patient demographics & baseline characteristics

Of the 198 patients who completed the core 6-week trial, 148 entered the 24-week extension study and received the study medication. Of these patients, 144 (61 % of the 235 patients included in the 6-week HRQoL analysis) with data on the PETiT scale or the SF-12 survey comprised the ITT population for the current analysis of HRQoL. Among the 141 patients with PETiT total and domain scores at core study baseline, 139 had PETiT scores at extension study baseline and 95 had PETiT scores at extension study endpoint. Among the 143 patients who had SF-12 scores at core study baseline and extension study baseline, 97 had SF-12 scores at extension study endpoint.

Table 1 presents a summary of the baseline characteristics for the ITT population. The majority of patients were male (63.2 %) and the mean (standard deviation [SD]) age at study entry was 42.6 (11.2) years. Close to one-quarter (23.6 %) of patients switched to lurasidone from quetiapine, 21.5 % from aripiprazole, 20.1 % from risperidone, 13.2 % from ziprasidone, and 9.0 % from olanzapine. The overall mean (SD) daily dose of lurasidone was 101.8 mg (77.6).

**Table 1:** Patient demographics and baseline characteristics at core study baseline

Parameter	
*N*	144
Mean age, years (SD)	42.6 (11.2)
Gender	
Male, *n* (%)	91 (63.2)
Race, *n* (%)	
Black or African American	92 (63.9)
White	46 (31.9)
Other	6 (4.2)
Mean age (SD) at initial onset of schizophrenia or schizoaffective disorder (years)	24.2 (9.4)
DSM-IV Schizophrenia subtype diagnosis, *n* (%)^a^	
295.10 Disorganized type	1 (0.7)
295.30 Paranoid type	70 (48.6)
295.60 Residual type	1 (0.7)
295.70 Schizoaffective disorder	55 (38.2)
295.90 Undifferentiated type	18 (12.5)
Pre-switch antipsychotic agent at study start, *n* (%)^b^	
Aripiprazole	31 (21.5)
Quetiapine	34 (23.6)
Risperidone	29 (20.1)
Ziprasidone	19 (13.2)
Olanzapine	13 (9.0)
Paliperidone	7 (4.9)
Iloperidone	2 (1.4)
Asenapine	2 (1.4)
First-generation antipsychotic^c^	17 (11.8)
Mean (SD) daily dose of lurasidone (mg)	101.8 (77.6)
Employment status, *n* (%)	*N* = 95^d^
Employed	14 (14.7)
Unemployed	81 (85.3)

*Abbreviations*: *DSM-IV* Diagnostic and Statistical Manual of Mental Disorders IV, *SD* standard deviation

^a^Patients could have more than one DSM-IV schizophrenia subtype of diagnosis

^b^Patients could be on multiple pre-switch antipsychotics

^c^First-generation antipsychotics included chlorpromazine, fluphenazine, haloperidol, perphenazine, and tiotixene

^d^Patients with both PETiT and employment status data

### PETiT assessment

#### Total and domain scores

**Fig. 2:**
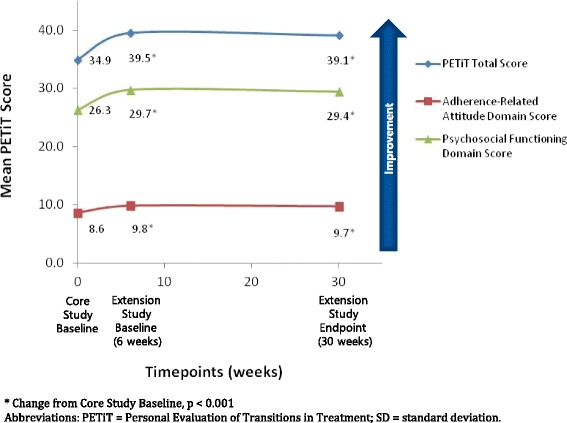
Mean Changes in PETiT total and domain scores in patients switched to long-term lurasidone therapy

At core study baseline, the PETiT scale was found to have high reliability based on a Cronbach’s alpha of 0.88. The mean (SD) PETiT total score for all lurasidone patients improved from 34.9 (9.3) at core study baseline to 39.5 (8.9) at extension study baseline and 39.1 (9.0) at extension study endpoint (Fig. 2; Table 2). This change in scores represented statistically significant improvements of 4.5 (7.9) and 5.1 (7.2) (both *p* < 0.001) at 6 weeks and after an additional 24 weeks of treatment, respectively. Mean (SD) changes from core baseline to extension study baseline and extension study endpoint were also significant in the domains of adherence-related attitude (1.2 [2.3] and 1.3 [2.5], respectively) and psychosocial functioning (3.3 [6.5] and 3.8 [5.8], respectively) (all *p* < 0.001) (Fig. 2; Table 2).

**Table 2:** Mean changes in PETiT total, domain, and subdomain scores in patients switched to lurasidone

		Core Study Baseline	Extension Study Baseline (6 weeks)	Extension Study Endpoint (end of additional 24 weeks of treatment)
	Number	141^a^	139^b^	95^c^
PETiT TotalScore	Mean Score (SD)	34.9 (9.3)	39.5 (8.9)	39.1 (9.0)
	Change from Core BL (SD)	--	4.5 (7.9)	5.1 (7.2)
	*p*-value	--	<0.001	<0.001
Adherence-related Attitude Domain Score (6 items)	Mean Score (SD)	8.6 (2.2)	9.8 (1.9)	9.7 (1.8)
	Change from Core BL (SD)	--	1.2 (2.3)	1.3 (2.5)
	*p*-value	--	<0.001	<0.001
Psychosocial Functioning Domain Score (24 items)	Mean Score (SD)	26.3 (8.1)	29.7 (7.8)	29.4 (8.0)
	Change from Core BL (SD)	--	3.3 (6.5)	3.8 (5.8)
	*p*-value	--	<0.001	<0.001
Social Functioning Subdomain (4 items)	Mean Score (SD)	3.8 (1.3)	4.0 (1.5)	3.9 (1.4)
	Change from Core BL (SD)	--	0.1 (1.4)	0.2 (1.3)
	*p*-value	--	0.534	0.361
Activity Subdomain (7 items)	Mean Score (SD)	7.8 (2.8)	8.7 (2.8)	8.5 (2.9)
	Change from Core BL (SD)	--	1.0 (2.6)	1.1 (2.6)
	*p*-value	--	<0.001	<0.001
Cognitive Subdomain (7 items)	Mean Score (SD)	8.2 (2.9)	9.3 (2.7)	9.3 (2.4)
	Change from Core BL (SD)	--	1.1 (2.3)	1.3 (2.1)
	*p*-value	--	<0.001	<0.001
Dysphoria Subdomain (6 items)	Mean Score (SD)	6.6 (2.5)	7.7 (2.2)	7.6 (2.6)
	Change from Core BL (SD)	--	1.1 (2.1)	1.2 (2.0)
	*p*-value	--	<0.001	<0.001

*Abbreviations*: *BL* baseline, *PETiT* Personal Evaluation of Transitions in Treatment, *SD* standard deviation

^a^Number of patients with PETiT total and domain scores at core study baseline

^b^Number of patients with PETiT total and domain scores at both core study baseline and extension study baseline

^c^Number of patients with PETiT total and domain scores at both core study baseline and extension study endpoint

Patients additionally showed significant improvements in three of the four subdomains of psychosocial functioning: scores for activity, cognitive, and dysphoria were higher at both extension study baseline and extension study endpoint (Table 2; all *p* < 0.001). Scores for social functioning remained comparable to those at core baseline.

### Outcome by prior antipsychotic

Prior antipsychotics received by at least 9 % of patients in the extension study included quetiapine, olanzapine, risperidone, aripiprazole, and ziprasidone. Patients who switched from any of these medications except olanzapine showed statistically significant improvements in the PETiT total score from core baseline to extension baseline (*p* ≤ 0.024) (Table 3). Subjects switched from quetiapine and aripiprazole maintained this significant difference at extension study endpoint (*p* ≤ 0.026). Improvements, some of which were significant, in the PETiT domains of adherence-related attitude and psychosocial functioning were also observed, particularly for subjects switching from quetiapine or aripiprazole. For subjects switched from olanzapine, increases in PETiT total and domain scores were observed from core study baseline through extension study endpoint (Table 3); however, these differences were either not statistically significant or significance could not be estimated as a result of small sample sizes.

**Table 3:** Mean changes in PETiT total and domain scores by prior medication in patients switched to long-term lurasidone therapy

		Quetiapine			Olanzapine			Risperidone			Aripiprazole			Ziprasidone		
		Core Study BL	Extension Study BL	Extension Study EP	Core Study BL	Extension Study BL	Extension Study EP	Core Study BL	Extension Study BL	Extension Study EP	Core Study BL	Extension Study BL	Extension Study EP	Core Study BL	Extension Study BL	Extension Study EP
PETiT Total Score	*N*	33^a^	32^b^	23^c^	12^a^	12^b^	8^c^	25^a^	25^b^	13^c^	28^a^	28^b^	22^c^	19^a^	19^b^	13^c^
	Mean Score (SD)	30.1 (8.0)	35.8 (9.6)	34.0 (6.6)	40.3 (11.8)	43.1 (11.1)	41.4 (12.1)	36.4 (9.9)	40.9 (8.5)	39.4 (11.9)	35.5 (7.6)	40.1 (8.1)	41 (7.7)	35.5 (8.2)	40.9 (7.4)	39.5 (8.6)
	Change from Core BL (SD)	--	5.3 (9.1)	3.0 (6.0)	--	1.6 (5.6)	3.1 (7.6)	--	5 (7.5)	8.4 (9.6)	--	4.4 (6.9)	5.5 (6.1)	--	5.4 (7.4)	5.3 (7.2)
	*p*-value	--	0.004	0.026	--	0.082	NE^d^	--	0.024	0.126	--	0.021	0.002	--	0.005	0.233
Adherence-related Attitude Domain Score	Mean Score (SD)	8.0 (2.2)	9.2 (2.3)	8.7 (2.0)	9.2 (2.4)	10.2 (1.7)	9.4 (1.7)	8.8 (2.1)	10.1 (1.7)	10.1 (2.2)	8.3 (2.1)	10.1 (1.7)	10.2 (1.4)	8.6 (2.0)	9.9 (1.9)	9.8 (1.9)
	Change from Core BL (SD)	--	1.3 (2.7)	0.9 (2.5)	--	0.9 (2.1)	0.6 (1.8)	--	1.3 (2.0)	1.8 (2.5)	--	1.7 (2.1)	1.8 (2.5)	--	1.3 (1.9)	1.2 (2.5)
	*p*-value	--	0.004	0.039	--	0.226	NE^d^	--	0.002	0.138	--	0.001	<0.001	--	0.059	0.119
Psychosocial Functioning Domain Score	Mean Score (SD)	22.1 (7.0)	26.6 (8.5)	25.3 (6.1)	31.1 (10)	32.8 (9.6)	32 (10.5)	27.6 (8.7)	30.8 (7.6)	29.4 (10.3)	27.3 (6.6)	30.0 (7.3)	30.8 (7.3)	26.9 (7.2)	31.1 (6.4)	29.7 (7.6)
	Change from Core BL (SD)	--	3.9 (7.5)	2.1 (4.4)	--	0.7 (4.3)	2.5 (6.9)	--	3.8 (6.6)	6.5 (8.0)	--	2.7 (5.8)	3.7 (5.4)	--	4.1 (6.2)	4.2 (5.3)
	*p*-value	--	0.011	0.083	--	0.200	NE^d^	--	0.058	0.131	--	0.081	0.013	--	0.003	0.228

*Abbreviations*: *BL* baseline, *EP* endpoint, *NE* Not estimable, *PETiT* Personal Evaluation of Transitions in Treatment, *SD* standard deviation

*Note*: Extension study BL = 6-week follow-up; extension study BL = 30-week follow-up

^a^Number of patients with PETiT total and domain scores at core study baseline

^b^Number of patients with PETiT total and domain scores at both core study baseline and extension study baseline

^c^Number of patients with PETiT total and domain scores at both core study baseline and extension study endpoint

^d^Algorithm from regression did not converge due to small population size; *p*-value is therefore not estimable

### Outcome by responder status

Responders to lurasidone (i.e., those having a ≥20 % improvement on the PANSS Total Score at Week 6 compared with core study baseline) showed significantly greater improvement in PETiT total (8.4 vs. 3.5, *p* = 0.01) and psychosocial functioning (6.4 vs. 2.5, *p* = 0.014) scores from core baseline to extension study baseline than nonresponders (Table 4). Though not significant, responders also showed greater improvement in PETiT total, adherence-related attitude domain, and psychosocial functioning domain scores than non-responders from core baseline to extension study endpoint. Responders demonstrated the greatest improvement in the PETiT total score from core baseline to extension study baseline; this improvement was sustained at the extension study endpoint (8.4 vs. 8.0). While non-responders had modest improvement in the PETiT total score from core baseline to extension study baseline, their HRQoL continued to improve over the course of treatment (i.e., at extension study endpoint) with lurasidone (3.5 vs. 4.1).

**Table 4:** Mean changes in PETiT total and domain scores by responder status

		Responders			Non-responders		
		Core Study BL	Extension Study BL	Extension Study EP	Core Study BL	Extension Study BL	Extension Study EP
PETiT Total Score	*N*	31^a^	31^b^	24^c^	110^a^	108^b^	71^c^
	Mean Score (SD)	32.5 (8.4)	41.0 (10.4)	40.5 (9.6)	35.6 (9.5)	39.1 (8.4)	38.6 (8.8)
	Change from Core BL (SD)	--	8.4 (8.5)	8.0 (6.5)	--	3.5 (7.4)	4.1 (7.1)
	*p*-value (between group)	--	0.010*	0.152**	--	--	--
Adherence-related Attitude Domain Score	Mean Score (SD)	8.1 (2.2)	10.1 (2.1)	10.0 (1.7)	8.8 (2.2)	9.7 (1.8)	9.6 (1.9)
	Change from Core BL (SD)	--	2.0 (2.2)	2.0 (2.2)	--	0.9 (2.3)	1.0 (2.5)
	*p*-value (between group)	--	0.051*	0.318ˣ**	--	--	--
Psychosocial Functioning Domain Score	Mean Score (SD)	24.5 (7.3)	30.9 (8.8)	30.6 (8.5)	26.8 (8.2)	29.4 (7.5)	29.0 (7.8)
	Change from Core BL (SD)	--	6.4 (7.0)	6.0 (5.5)	--	2.5 (6.1)	3.0 (5.7)
	*p*-value (between group)	--	0.014*	0.220**	--	--	--

*Note*: Extension study BL = 6-week follow-up; extension study EP = end of additional 24 weeks of treatment

*Abbreviations*: *BL* baseline, *EP* endpoint, *PETiT* Personal Evaluation of Transitions in Treatment, *SD* standard deviation

^a^Number of patients with PETiT total and domain scores at core study baseline

^b^Number of patients with PETiT total and domain scores at both core study baseline and extension study baseline

^c^Number of patients with PETiT total and domain scores at both core study baseline and extension study endpoint

*P-value comparing mean changes in PETiT total/domain scores from extension study baseline to core study baseline between responders and non-responders

**P-value comparing mean changes in PETiT total/domain scores from extension study endpoint to core study baseline between responders and non-responders

### SF-12 assessment

#### Summary scores

At core study baseline, the SF-12 summary scales were found to have adequate reliability based on a Cronbach’s alpha of 0.76 for the PCS and 0.74 for the MCS. The SF-12 results showed that health status improved or remained stable between core baseline, extension baseline, and the end of an additional 24 weeks of treatment (extension study endpoint; Table 5). Changes on the MCS score were statistically significant between the core and extension baselines (mean [SD]: 4.8 [11.6], *p* < 0.001) and between core baseline and extension study endpoint (6.3 [10.3], *p* < 0.001). PCS scores were maintained between core baseline and extension study baseline and showed significant improvement from core baseline to extension study endpoint (2.6 [7.9], *p*=0.001).

**Table 5:** Mean changes in SF-12 PCS and MCS scores

		Core Study Baseline	Extension Study Baseline (6 weeks)	Extension Study Endpoint (end of additional 24 weeks of treatment)
	N	143^a^	143^b^	97^c^
PCS Score	Mean Score (SD)	46.5 (10.6)	46.3 (10.1)	48.6 (9.4)
	Change from Core Baseline (SD)	--	−0.3 (8.4)	2.6 (7.9)
	*p*-value	--	0.629	0.001
MCS Score	Mean Score (SD)	41.8 (11.1)	46.5 (11.2)	47.3 (9.6)
	Change from Core Baseline (SD)	--	4.8 (11.6)	6.3 (10.3)
	*p*-value	--	<0.001	<0.001

*Abbreviations*: *MCS* Mental Component Summary, *PCS* Physical Component Summary, *SD* standard deviation, *SF-12* Short Form-12

^a^Number of patients with SF-12 PCS and MCS scores at core study baseline

^b^Number of patients with SF-12 PCS and MCS scores at both core study baseline and extension study baseline

^c^Number of patients with SF-12 PCS and MCS scores at both core study baseline and extension study endpoint

#### Outcome by prior antipsychotic

Analysis of subjects by prior antipsychotic agent also showed improvement or maintenance of health status following long-term switch to lurasidone, particularly on the MCS (Table 6). Subjects switched from aripiprazole or ziprasidone showed statistically significant improvements on the MCS score between core and extension baseline (mean change [SD]: 3.7 [8.4] and 7.7 [10.5], respectively; both *p* = 0.039). Similarly, subjects switched from quetiapine showed significant improvement on the MCS score between core baseline and extension study endpoint (Week 30) (5.3 [10.9], *p* = 0.030). In general, PCS scores remained stable from core baseline through the end of the study regardless of preswitch antipsychotic agents.

**Table 6:** Mean changes in SF-12 PCS and MCS scores by prior medication in patients switched to long-term lurasidone therapy

		Quetiapine			Olanzapine			Risperidone			Aripiprazole			Ziprasidone		
		Core Study BL	Extension Study BL	Extension Study EP	Core Study BL	Extension Study BL	Extension Study EP	Core Study BL	Extension Study BL	Extension Study EP	Core Study BL	Extension Study BL	Extension Study EP	Core Study BL	Extension Study BL	Extension Study EP
Physical Component Summary Score	N	30^a^	30^b^	25^c^	13^a^	13^b^	7^δ^	27^a^	27^b^	14^c^	31^a^	31^b^	20^c^	17^a^	17^b^	14^c^
	Mean Score (SD)	45.1 (11.5)	42.9 (10.1)	44.4 (9.8)	50.3 (11.1)	49.5 (10.2)	49.3 (7.7)	46.6 (9.4)	49.7 (8.6)	49.5 (6.5)	46.2 (11.1)	45.5 (11.0)	51.1 (9.1)	47.7 (10.5)	45.7 (10.6)	48.3 (9.7)
	Change from Core BL (SD)	--	−1.0 (9.5)	0.9 (7.4)	--	−0.2 (5.0)	1.2 (10.1)	--	1.9 (9.5)	2.0 (7.6)	--	−0.9 (7.8)	4.4 (7.7)	--	−1.8 (6.3)	2.5 (10.0)
	*p*-value	--	0.555	0.524	--	0.239	NE^d^	--	0.302	0.055	--	0.712	0.047	--	0.166	0.497
Mental Component Summary Score	N	30	30	25	13	13	7	27	27	14	31	31	20	17	17	14
	Mean Score (SD)	38.9 (11.1)	43.0 (12.3)	42.7 (9.3)	45.0 (12.1)	52.0 (11.7)	47.9 (10.9)	42.0 (12.1)	46.3 (11.0)	50.2 (9.9)	42.2 (8.1)	46.1 (9.2)	47.8 (9.3)	40.7 (8.8)	47.6 (9.6)	47.4 (8.9)
	Change from Core BL (SD)	--	4.8 (14.1)	5.3 (10.9)	--	7.8 (9.1)	3.0 (5.3)	--	4.7 (12.1)	10.2 (14.4)	--	3.7 (8.4)	6.0 (9.3)	--	7.7 (10.5)	5.8 (9.5)
	*p*-value	--	0.103	0.030	--	0.224	NE^d^	--	0.057	<0.001	--	0.039	<0.001	--	0.039	0.035

*Note*: Extension study BL = 6-week follow-up; extension study BL = 30-week follow-up

*Abbreviations*: *BL* baseline, *EP* endpoint, *NE* Not estimable, *MCS* Mental Component Summary, *PCS* Physical Component Summary, *SD* standard deviation, *SF-12* Short Form 12

^a^Number of patients with SF-12 PCS/MCS scores at core study baseline

^b^Number of patients with SF-12 PCS/MCS scores at both core study baseline and extension study baseline

^c^Number of patients with SF-12 PCS/MCS scores at both core study baseline and extension study endpoint

^d^Algorithm from regression did not converge due to small population size; *p*-value is therefore not estimable

#### Outcome by responder status

Responders to lurasidone demonstrated greater improvement on the MCS score than non-responders from core baseline to extension study baseline (mean change: 11.3 vs. 3.0; *p* = 0.001). There was no statistically significant difference in improvement on the PCS score between responders and non-responders, both from core baseline to extension baseline and from core baseline to extension study endpoint (Table 7).

**Table 7:** Mean Changes in SF-12 PCS and MCS Scores by Responder Status

		Responders			Non-responders		
		Core Study BL	Extension Study BL	Extension Study EP	Core Study BL	Extension Study BL	Extension Study EP
	*N*	31^a^	31^b^	25^c^	112^a^	112^b^	72^c^
PCS score	Mean Score (SD)	46.4 (11.4)	47.4 (10.7)	48.9 (9.9)	46.6 (10.4)	46.0 (9.9)	48.4 (9.3)
	Change from Core BL (SD)	--	0.6 (9.6)	2.7 (6.7)	--	−0.5 (8.0)	2.5 (8.3)
	*p*-value (between group)	--	0.239*	0.643**	--	--	--
MCS score	Mean Score (SD)	38.9 (8.6)	49.6 (11.5)	48.5 (9.0)	42.6 (11.6)	45.6 (11.0)	46.9 (9.8)
	Change from Core BL (SD)	--	11.3 (10.1)	10.0 (10.1)	--	3.0 (11.3)	5.0 (10.1)
	*p*-value (between group)	--	0.001*	0.280**	--	--	--

*Note*: Extension study BL = 6-week follow-up; extension study BL = end of additional 24 weeks of treatment

*Abbreviations: BL* baseline, *EP* endpoint, *NE* Not estimable, *MCS* Mental Component Summary, *PCS* Physical Component Summary, *SD* standard deviation, *SF-12* Short Form 12

^a^Number of patients with SF-12 PCS/MCS scores at core study baseline

^b^Number of patients with SF-12 PCS/MCS scores at both core study baseline and extension study baseline

^c^Number of patients with SF-12 PCS/MCS scores at both core study baseline and extension study endpoint

*P-value comparing mean changes in SF-12 PCS/MCS scores from extension study baseline to core study baseline between responders and non-responders

**P-value comparing mean changes in SF-12 PCS/MCS scores from extension study endpoint to core study baseline between responders and non-responders

## Discussion

The current study examined the long-term effects of lurasidone on HRQoL among clinically stable yet symptomatic patients with schizophrenia or schizoaffective disorder switched to lurasidone. The majority of patients showed improvements in HRQoL on the PETiT total and SF-12 MCS scores from core baseline to extension study baseline (6 weeks) and to extension study endpoint (end of additional 24 weeks of treatment). Sustained improvements were also demonstrated on the PETiT domains of adherence-related attitude and psychosocial functioning and the psychosocial subdomains of activity, cognition, and dysphoria. Given that psychosocial functioning is a key outcome measure in understanding the effects of a successful treatment in schizophrenia [57], this finding of sustained improvement during lurasidone therapy may have significant clinical implications.

Subgroup analyses of the results by prior antipsychotic and responder status further showed improvement of HRQoL and functioning during both short- and long-term treatment with lurasidone. Patients who switched from any of the analyzed antipsychotics (quetiapine, olanzapine, risperidone, aripiprazole, ziprasidone) generally showed improvements from core baseline to extension study baseline and/or extension study endpoint. These improvements were most likely to be statistically significant in patients switched from quetiapine or aripiprazole. While the larger sample size of patients switching from these therapies may have contributed to this finding, it is reasonable to hypothesize that the reduced risk of sedation [58, 59] and agitation [60, 61] associated with lurasidone may have played a role. Responders to lurasidone generally showed greater improvements on the PETiT scale and SF-12 scores (MCS) than non-responders. The lack of statistical significance between these groups for changes from baseline to study endpoint may relate to small sample sizes. Notably, while non-responders had only modest improvement on the PETiT total score during short-term treatment, their HRQoL continued to improve during longer-term therapy. This observation may arise because a subset of these patients responded to treatment at a slower pace (i.e., were “late responders”).

Guidelines from the National Institute for Health and Care Excellence (NICE) and the American Psychiatric Association emphasize the importance of choosing the most appropriate antipsychotic drug and formulation based on a patient’s needs and characteristics, rather than relying solely on the main recognized properties of different drug classes [62, 63]. A patient-centered approach to treatment selection may contribute to increased adherence to therapy while reducing the risk of adverse events, relapse, rehospitalization, and ultimately poor HRQoL [52, 64, 65].

In addition to symptoms and treatment type, a schizophrenia patient’s subjective response, perception, and attitude (i.e., how they perceive their clinical response and/or adverse events) to therapy are suggested to play key roles in HRQoL, adherence, and other long-term outcomes [14, 66–71]. Adherence to antipsychotic therapy is an ongoing problem in the management of schizophrenia, with an estimated 40 % of patients being partially or fully non-adherent [16, 71]. One-year data from the Cost Utility of the Latest Schizophrenia Antipsychotic drugs in Schizophrenia (CUtLASS) randomized controlled trial showed that improvements in adherence led to greater quality of life [14]; similarly, 3 year results from the European Schizophrenia Outpatients Health Outcomes (EU-SOHO) study demonstrated that continuous antipsychotic treatment was associated with important HRQoL benefits [17]. Other studies indicate that improved adherence can lead to greater treatment efficacy, thereby reducing symptoms and the implications of inadequate therapy such as relapse and hospitalization [13, 14, 16, 22, 52, 64, 65, 72, 73]. Given the significant clinical and economic burden associated with psychiatric relapses and hospitalizations in schizophrenia, the current study’s finding of improved or maintained adherence-related attitude after switching to lurasidone is of interest and may have implications for future research on treatment adherence with this therapy.

Switching antipsychotic therapies has been previously found to lead to improvements in schizophrenia. A study by Roussidis and colleagues showed that switching a patient’s antipsychotic medication for reasons related to lack of efficacy and/or tolerability was associated with a significantly improved clinical benefit and increased adherence to treatment [74]. Similarly, studies of patients switching to olanzapine [28], quetiapine [29, 34], ziprasidone [33, 75], aripiprazole [76], long-acting injectable risperidone [32, 77], or paliperidone [36] have reported improvements in cognitive function, psychotic symptoms, and/or tolerability during up to one year of treatment. However, the current analysis is the only switch study to examine long-term changes in subjective responses, tolerability, adherence-related attitude, and HRQoL using the disease-specific PETiT assessment.

Overall, the results of the current study demonstrate that switching to lurasidone after inadequate response to current antipsychotic therapy is associated with improvements in HRQoL and health status, particularly in responders to treatment. While HRQoL improvement was observed among schizophrenia patients having switched to lurasidone in the current analysis, the authors recognize several limitations of the study. The study was an open-label trial in which treatment was not masked to either patients or physicians. The results could reflect the passage of time, as the lack of a parallel control group precluded study of the impact of discontinuation of lurasidone. Moreover, patients who discontinued the study may have differed from those who completed the study. As with any self-reported outcome, patient responses on the PETiT and SF-12 may have been biased by patient’s expectation, recall issues, or other aspects of the treatment experience. Further, given the small sample sizes for the prior antipsychotic and responder analyses, interpretation of these results may warrant caution. Finally, evaluation of PETiT and SF-12 scores at multiple time points throughout the trial rather than only at baseline, 6 weeks, and after an additional 24 weeks of treatment would have provided a more complete picture of lurasidone-induced changes in HRQoL. Nevertheless, this switch trial provides important guidance on clinical practice concerning switching patients to lurasidone in the context of long-term HRQoL benefits.

## Conclusions

In conclusion, the results of this follow-up study demonstrated that stable yet symptomatic patients with schizophrenia or schizoaffective disorder who switched to lurasidone from other antipsychotics experienced long-term improvements in HRQoL. When healthcare providers continue to work with schizophrenia patients to optimize treatment, long-term HRQoL gains can be achieved. To our knowledge, this study is the first to assess long-term HRQoL outcomes after switch to lurasidone, is one of only few antipsychotic switch studies with a follow-up duration ≥6 months, and is the only switch study to use the validated, disease-specific PETiT scale. Further research is warranted to understand the impact of lurasidone-related improvements in HRQoL on medication adherence, relapse and rehospitalization rates, employment status, and overall costs to the health care system.

## Abbreviations

AE, adverse event; ANCOVA, analysis of covariance; BL, baseline; CDSS, calgary depression scale for schizophrenia; CGI-S, clinical global impressions-severity; CUtLASS, cost utility of the latest schizophrenia antipsychotic drugs in schizophrenia; DSM, diagnostic and statistical manual of mental disorders; EP, endpoint; EU-SOHO, european schizophrenia outpatients health outcomes; HRQoL, health-related quality of life; ITT, intention to treat; MCS, mental component score; NE, not estimable; NICE, national institute for health and care excellence; PANSS, positive and negative syndrome scale; PCS, physical component scale; PETiT, personal evaluation of transitions in treatment; SD, standard deviation; SF-12, short-form 12; TEAE, treatment-emergent adverse event
